# Dietary Adjuvanticity in the Modern Plant Exposome: Implications for Immune-Mediated Inflammatory Diseases

**DOI:** 10.3390/nu18162662

**Published:** 2026-08-14

**Authors:** Zsolt Barta, Edit Posta, Eva Gyarmati, Judit Baranyi, Istvan Fekete, Eva Zold

**Affiliations:** 1 Department of Infectology, Faculty of Medicine, University of Debrecen, Bartok Bela U 2-26, 4031 Debrecen, Hungary; 2 Doctoral School of Clinical Immunology and Allergology, Faculty of Medicine, University of Debrecen, Nagyerdei Krt. 98, 4032 Debrecen, Hungary; 3 Doctoral School of Health Sciences, University of Debrecen, Nagyerdei Krt. 98, 4032 Debrecen, Hungary; 4 Institute of Food Technology, Faculty of Agricultural and Food Sciences and Environmental Management, University of Debrecen, Boszormenyi Ut 138, 4032 Debrecen, Hungary; 5 Department of Clinical Immunology, Institute of Internal Medicine, Faculty of Medicine, University of Debrecen, Moricz Zsigmond Krt. 22, 4032 Debrecen, Hungary; zold.eva@med.unideb.hu

**Keywords:** dietary adjuvanticity, plant exposome, plant defence molecules, gut barrier, microbiota, host immunity, ASIA, immune-mediated inflammatory diseases, coeliac disease, inflammatory bowel disease

## Abstract

Immune-mediated inflammatory diseases (IMIDs) arise from interactions among genetic susceptibility, epithelial barrier function, microbiota, diet, and other environmental exposures. Modern diets influence mucosal immunity not only through fibre intake, food processing, and microbiota composition, but also through a less explored exposure layer: plant-derived molecules with potential immune activity. Crop breeding, intensive agriculture, global trade, gluten-free substitutes, and plant-based food technologies have changed the spectrum, dose, concentration, and matrix in which plant defence proteins, antinutritional factors, endogenous toxicants, and novel plant antigens reach the intestinal surface. In this structured, hypothesis-generating narrative review, we propose dietary adjuvanticity as a mechanistic framework for considering how selected food-derived molecules may amplify mucosal immune responsiveness, modify antigen presentation, disturb barrier function, or lower tolerance thresholds without necessarily acting as classical autoantigens. The framework differs from general food-derived immunomodulation, nutritional exposomics, and diet-microbiota-host interaction models by focusing specifically on adjuvant-like immune amplification at the intestinal mucosa. The ASIA concept is used only in Shoenfeld’s functional sense, as an analogy for exogenous immune amplification through innate activation, danger signalling, bystander activation, epitope spreading, and loss of tolerance in susceptible hosts; it is not applied as a dietary diagnosis. Wheat amylase-trypsin inhibitors, gluten epitopes, lectins, potato glycoalkaloids, saponins, quinoa prolamins, emerging legume proteins, L-canavanine, and tolerance-promoting plant substrates are discussed with explicit separation of established clinical evidence, strong mechanistic evidence, preclinical/ex vivo evidence, and speculative disease-modifier hypotheses. Overall, plant-derived exposures are best interpreted as potential modifiers within the IMID exposome, not as primary causes of autoimmunity. Testing this model will require defined exposures, food-matrix and processing studies, biomarkers of barrier and immune activation, patient stratification, and controlled human studies.

## 1. Introduction

IMIDs comprise a broad and heterogeneous disease spectrum that includes coeliac disease, IBD, allergic disorders, eosinophilic gastrointestinal disease, and several systemic autoimmune conditions. These disorders do not arise from a single cause. Their pathogenesis usually requires convergence of genetic susceptibility, epithelial or tissue barrier vulnerability, innate and adaptive immune dysregulation, microbiota-related signals, and environmental exposures. A large population-based analysis of 22 million individuals in the United Kingdom showed that autoimmune disorders collectively affect a substantial proportion of the population and that incidence varies by disease, sex, age, socioeconomic status, season, and region [1]. IBD also represents a growing global burden, with population-based studies documenting substantial geographic variation and increasing prevalence in many regions [2]. Coeliac disease is similarly distributed worldwide, with pooled seroprevalence and biopsy-confirmed prevalence estimates supporting its importance as a common immune-mediated enteropathy [3]. These epidemiological patterns strengthen the rationale for studying modifiable environmental factors, including diet, at the gut-immune interface.

Genetic predisposition provides the disease-specific framework in which environmental exposures act. In coeliac disease, HLA-DQ2/DQ8 permits presentation of deamidated gluten peptides and defines a clear antigen-specific disease model [4,5,6]. In IBD, variants affecting microbial sensing, autophagy, epithelial defence, and barrier repair, including NOD2- and ATG16L1-related pathways, interact with the intestinal microbiota and the luminal environment [7,8,9]. In systemic IMIDs, susceptibility genes influence antigen presentation, cytokine circuits, and immune tolerance, but genetics alone cannot explain temporal increases, geographic gradients, urbanization effects, or disease flares. This sequence is important: dietary exposures are considered here as modifiers acting on genetically and immunologically susceptible backgrounds, not as universal or independent causes of IMIDs.

Diet is among the most plausible and modifiable components of the immune exposome. Most current discussions focus on Western dietary patterns, ultra-processed foods, excess fat and sugar, low fibre intake, emulsifiers, salt, additives, and microbiome disruption [7,9,10]. Recent European gastroenterology policy documents also describe unhealthy diets and ultra-processed-food-dominated environments as preventable drivers of digestive diseases and digestive cancers. The UEG nutrition paper links high ultra-processed food exposure to dysbiosis, intestinal inflammation, and impaired barrier function, while emphasizing obesity, metabolic dysfunction, food insecurity, and unequal access to minimally processed foods [11]. These public-health observations do not identify specific plant molecules as disease causes. They provide a broader rationale for examining how modern food systems reshape the diet-gut barrier-microbiota-immune interface.

We use the term modern plant exposome to describe an underexplored layer of dietary exposure. Plants are more than sources of energy, fibre, and micronutrients. They also contain storage and structural proteins, enzyme inhibitors, lectins, saponins, glycoalkaloids, glucosinolates, furanocoumarins, non-protein amino acids, and many secondary metabolites involved in defence, reproduction, and stress adaptation. In human nutrition, some are described as antinutritional factors, toxicants, allergens, or bioactive compounds. Their biological effects depend on dose, food matrix, processing, microbial metabolism, and host susceptibility [12,13,14,15]. Modern agriculture, global supply chains, gluten-free substitutes, and plant-protein technologies have altered not only which plants are eaten, but also how often, how concentrated, and in which patient populations they are consumed.

This review links three areas that are usually discussed separately: the ASIA concept, changes in crops and food systems, and mucosal immunology in IMIDs. The ASIA literature provides a conceptual vocabulary for exogenous immune amplification, but not a diagnostic label for dietary reactions. We do not argue that plant foods cause ASIA syndrome, nor that modern crops are primary causes of autoimmunity. Instead, Shoenfeld’s functional interpretation of adjuvants is used to ask a narrower and testable question: can selected plant-derived molecules behave as mucosal, dietary adjuvant-like exposures in susceptible hosts? At the intestinal surface, such molecules could theoretically amplify innate immune activation, reshape antigen presentation, promote bystander inflammation, or lower tolerance thresholds without serving as disease-specific autoantigens.

The central knowledge gap is therefore not whether plant foods are generally beneficial or harmful. The question is whether defined plant-derived exposures can act as context-dependent disease modifiers within the IMID exposome. This distinction preserves the established benefits of plant-rich, minimally processed dietary patterns while allowing specific molecules, matrices, and processing conditions to be evaluated mechanistically.

### 1.1. What This Review Adds

This review contributes five linked points. First, it defines the modern plant exposome as a distinct nutritional exposure layer within IMID research. Second, it distinguishes dietary antigenicity from dietary adjuvanticity: some food-derived molecules are disease-defining antigens, whereas others may act primarily as immune amplifiers. Third, it uses Shoenfeld’s functional adjuvant concept only as a mechanistic analogy and explicitly avoids transferring the ASIA diagnosis to food reactions. Fourth, it places the gut barrier, microbiota, and gut-associated immune system at the interface through which these exposures may modify host immunity. Fifth, it separates established clinical evidence from strong mechanistic evidence, preclinical/ex vivo evidence, and hypothesis-generating extensions, so that the framework remains useful without encouraging unsupported dietary restrictions.

### 1.2. Scope and Objectives

The objectives are to define dietary adjuvanticity; clarify how it differs from existing concepts such as food-derived immunomodulation, nutritional exposomics, mucosal immune activation, and diet-microbiota-host interactions; summarize candidate plant-derived exposures; distinguish levels of evidence; discuss selected intestinal and extraintestinal disease contexts; and identify research priorities required to validate or refute the hypothesis.

## 2. Methods

### 2.1. Review Design

This article was designed as a structured narrative, hypothesis-generating review. It was not intended to calculate pooled risk estimates or provide an exhaustive systematic inventory of all publications. Instead, it integrates mechanistic, clinical, preclinical, food-science, toxicological, allergological, microbiome, and conceptual evidence relevant to Shoenfeld’s functional interpretation of adjuvants, dietary adjuvanticity, modern plant-derived exposures, and IMIDs. The review prioritizes biological plausibility, translational relevance, and conceptual coherence while explicitly identifying where evidence is established, mechanistic, preliminary, or speculative.

### 2.2. Literature Search Strategy

Targeted searches were conducted in PubMed/MEDLINE and Google Scholar from 1 January 2000 to 30 June 2026. Older studies were included when they represented seminal mechanistic or toxicological observations, such as early glycoalkaloid, lectin, or L-canavanine reports. Search terms combined concepts related to ASIA syndrome, Shoenfeld syndrome, adjuvants, dietary adjuvanticity, plant defence molecules, antinutrients, wheat amylase-trypsin inhibitors, gluten, coeliac disease, non-coeliac wheat sensitivity, IBD, lectins, glycoalkaloids, saponins, quinoa prolamins, L-canavanine, lupin and pea allergy, plant-based meat alternatives, novel foods, gut barrier, microbiome, oral tolerance, ultra-processed foods, food processing, food systems, and IMIDs. Crop-science and food-technology sources were included when they clarified breeding, processing, food matrices, or compositional changes in plant foods. The search did not target a single age group; paediatric, adult, older-adult, animal, ex vivo, and in vitro data were included when relevant to the conceptual framework. The term fast food was not used as a primary search construct; where relevant, it was considered under the broader categories of ultra-processed foods, food matrices, and modern food-system exposures.

### 2.3. Inclusion and Exclusion Criteria

Sources were included when they addressed at least one of the following domains: the ASIA concept and the functional meaning of adjuvants; plant-derived molecules with immune, barrier-modifying, antinutritional, allergenic, toxicological, or microbiome-modulating activity; mechanisms linking diet with pattern-recognition receptor signalling, epithelial integrity, microbiome function, microbial metabolism, or oral tolerance; clinical or preclinical links with coeliac disease, NCWS, IBD, food allergy, eosinophilic oesophagitis, or selected systemic IMIDs; changes in exposure driven by crop breeding, agricultural intensification, globalization, gluten-free products, or plant-protein isolates; and food-processing or food-technology studies relevant to reducing or transforming immune-active compounds.

Sources were excluded when they were unrelated to plant-derived dietary exposures, discussed only generic dietary advice without mechanistic relevance, lacked accessible methodological detail, focused solely on non-dietary adjuvants without conceptual relevance, or made unsupported claims that could not be linked to peer-reviewed mechanistic, clinical, preclinical, or food-technology evidence. Case reports and case series were used only to illustrate mechanisms or emerging exposures and not as proof of causality.

### 2.4. Evidence Synthesis and Appraisal

Because the literature spans heterogeneous disciplines, evidence was synthesized narratively and grouped by mechanistic relevance to IMIDs. Studies were assigned to four descriptive evidence levels. Established clinical evidence was used when disease definitions, diagnostic criteria, and therapeutic implications are broadly accepted, as in gluten-driven coeliac disease and IgE-mediated allergy to defined plant proteins. Strong mechanistic evidence was used when reproducible experimental data identify a plausible receptor, cell type, pathway, or tissue mechanism, such as ATI-TLR4-related myeloid activation. Preclinical or ex vivo evidence was used when findings derive mainly from animal models, organoids, epithelial/immune co-cultures, or patient-derived ex vivo assays. Speculative or hypothesis-generating evidence was used when the argument is based on biological plausibility, indirect disease models, or extrapolation from related pathways.

No formal GRADE, PRISMA-based risk-of-bias assessment, or meta-analysis was performed, because the aim was conceptual synthesis rather than intervention-effect estimation. This limits the strength and reproducibility of causal conclusions. Accordingly, claims in the manuscript are framed as established only where supported by clinical consensus or direct human evidence; otherwise, they are described as mechanistic, preclinical, associative, or hypothesis-generating.

### 2.5. Conceptual Use of the Asia Framework

The ASIA framework is used analogically and mechanistically, not diagnostically. The review does not propose that plant-derived dietary molecules are established causes of ASIA syndrome. Rather, Shoenfeld’s broad functional view of adjuvants is used to frame the possibility that selected food-derived molecules may amplify mucosal immune responses, alter antigen presentation, or reduce tolerance thresholds in susceptible hosts. The framework should not be used to diagnose ASIA from dietary symptoms, equate food intolerance with autoimmunity, or imply that plant-derived exposures are generally harmful. Its value is narrower: it helps define how selected molecules might behave as adjuvant-like modifiers of mucosal immune responsiveness.

### 2.6. Use of Generative AI Tools

ChatGPT (OpenAI; GPT-5.5 Thinking) was used to support language editing, stylistic refinement, manuscript structuring, and preparation/refinement of conceptual figures. The tool was not used to generate original scientific data, perform data analysis, conduct independent literature selection, draw independent scientific conclusions, or replace authorial interpretation. All AI-assisted text and figure-related suggestions were critically reviewed, edited, and approved by the authors, who take full responsibility for the content of the manuscript.

## 3. Asia as an Analogy for Environmental Immune Stimulation

Autoimmune/inflammatory syndrome induced by adjuvants (ASIA), or Shoenfeld’s syndrome, was introduced to describe immune-mediated manifestations reported after exposure to external stimuli with adjuvant properties [16]. Classical examples include silicone implants, vaccine adjuvants, mineral oils, and other immune-stimulating materials [17,18,19,20,21,22,23]. Clinical spectrum reviews and proof-of-concept animal studies have expanded the discussion to Sjogren-like phenotypes, experimental models, commercial sheep, and vaccine-autoimmunity debates [24,25,26,27]. The unifying idea is not a single antigen, but a permissive inflammatory context in which innate activation, antigen presentation, bystander activation, molecular mimicry, epitope spreading, and eventual loss of tolerance may converge.

The ASIA concept remains scientifically debated. Concerns include diagnostic specificity, heterogeneous exposures, variable latency, incomplete causal inference, and reliance on symptom-based criteria [19,20,22,23]. These concerns are directly relevant to dietary applications because they argue against creating a new ASIA-like food diagnosis. They do not, however, invalidate the narrower mechanistic question of whether exogenous exposures can amplify immune responses in susceptible hosts. This review therefore uses ASIA only as a source of conceptual language for exogenous immune amplification, while rejecting diagnostic overextension.

### Shoenfeld’s Functional Interpretation of Adjuvants

In Shoenfeld’s formulation, an adjuvant can be understood functionally rather than only as a licensed vaccine component. In this broader view, an adjuvant is an exogenous factor capable of strengthening immune responses. It does not need to be the disease-defining antigen. It may instead create an immune-amplifying environment through danger signalling, pattern-recognition receptor activation, maturation of antigen-presenting cells, cytokine release, inflammasome activation, bystander activation, molecular mimicry, epitope spreading, or polyclonal lymphocyte activation [16,18,19,20].

This broader interpretation provides the conceptual basis for dietary adjuvanticity. Plant-derived molecules are not implanted or injected adjuvants and should not be interpreted diagnostically as ASIA triggers. Nevertheless, they repeatedly contact the intestinal epithelium, mucus layer, microbiota, and gut-associated immune system. If such molecules intensify innate signalling, modify antigen presentation, disrupt epithelial integrity, or reduce tolerance thresholds, they may functionally resemble mucosal adjuvant-like exposures. Wheat ATIs are the clearest example, because they are not the classical coeliac antigen yet can activate myeloid cells through TLR4-related signalling and intensify intestinal inflammation [28,29,30].

## 4. Dietary Adjuvanticity: Definition, Originality, and Mechanistic Boundaries

We define dietary adjuvanticity as the capacity of selected food-derived molecules to amplify mucosal immune responses, lower tolerance thresholds, or enhance inflammatory signalling without necessarily acting as classical autoantigens. The term is narrower than general food intolerance and broader than IgE-mediated food allergy. It describes immune-modulating properties at the interface between the intestinal epithelium, the gut-associated immune system, the microbiota, and the luminal dietary environment.

**Figure 1:**
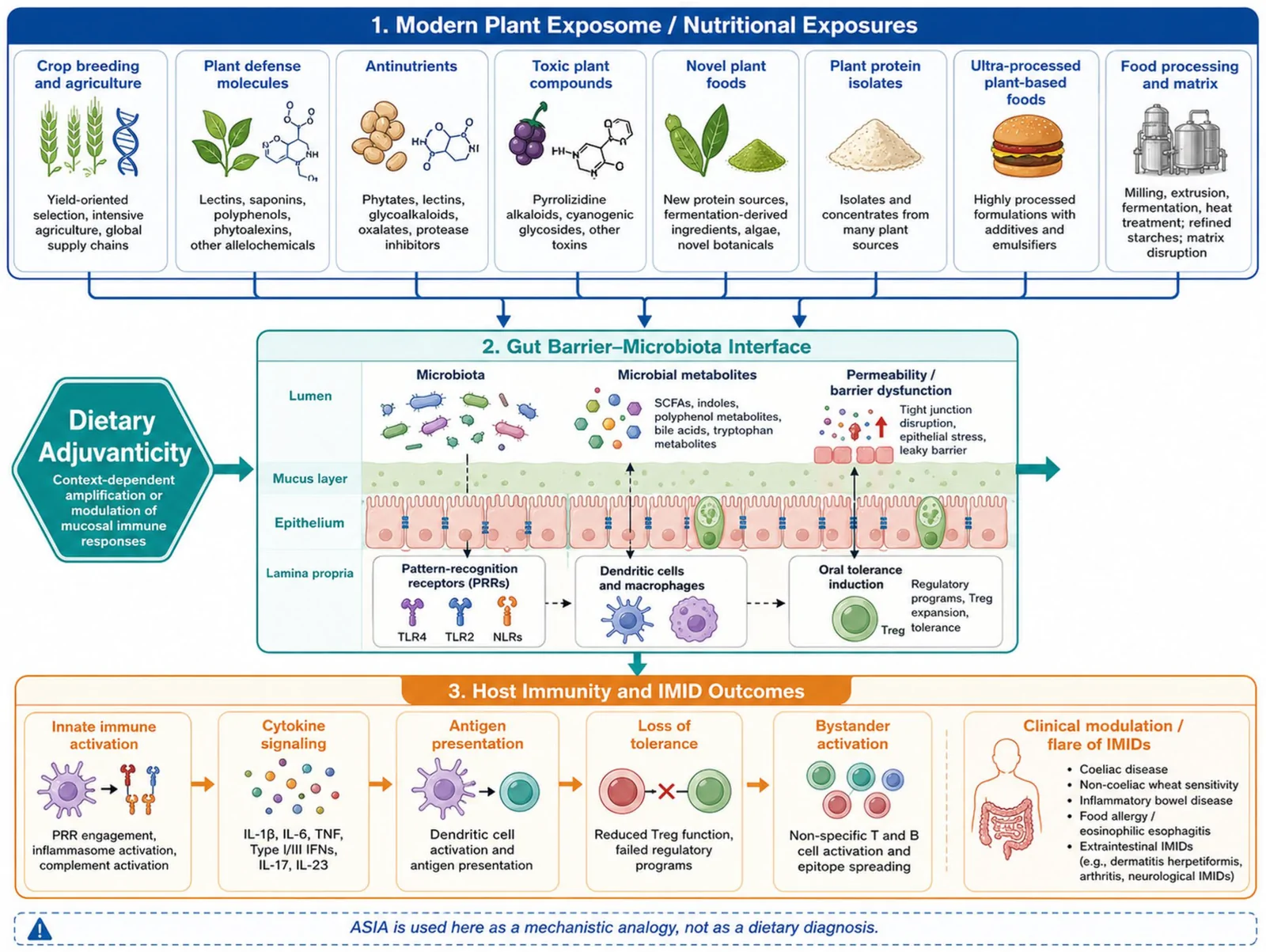
Conceptual model of dietary adjuvanticity at the diet-microbiota-host immunity interface. The modern plant exposome may influence the intestinal barrier, microbiota, pattern-recognition receptor signalling, antigen presentation, and oral tolerance. ASIA is used only as a mechanistic analogy for exogenous immune amplification, not as a dietary diagnosis.

These boundaries are summarized in Figure 1. Food-derived immunomodulation is a broad term that includes beneficial, neutral, or adverse immune effects of nutrients, fibres, metabolites, and bioactive compounds. Nutritional exposomics maps dietary exposures and their biomarkers but does not necessarily specify immune amplification. Mucosal immune activation describes the tissue response but not the dietary property that may generate it. Diet-microbiota-host interaction models emphasize ecological and metabolic pathways. Dietary adjuvanticity overlaps with these fields but focuses specifically on adjuvant-like amplification: the ability of a defined dietary molecule or matrix to intensify immune responsiveness to itself, to co-delivered antigens, or to microbial/damage signals in a susceptible mucosal context.

Several mechanisms are biologically plausible. Plant-derived molecules may impair epithelial barrier integrity and allow luminal antigens or microbial products to reach the lamina propria more easily [7,8]. They may activate innate immune receptors on epithelial cells, macrophages, or dendritic cells, as shown most clearly for wheat ATIs through TLR4-related signalling [28,29]. They may reshape the microbiota, altering mucosal immune tone through short-chain fatty acids, bile acids, tryptophan metabolites, and inflammatory thresholds [7,9,30]. They may also influence oral tolerance by changing antigen dose, antigen form, processing, presentation, or co-stimulatory context. In genetically susceptible hosts, dietary adjuvanticity may intersect with disease-specific pathways rather than act alone(Table 1).

**Figure 2:**
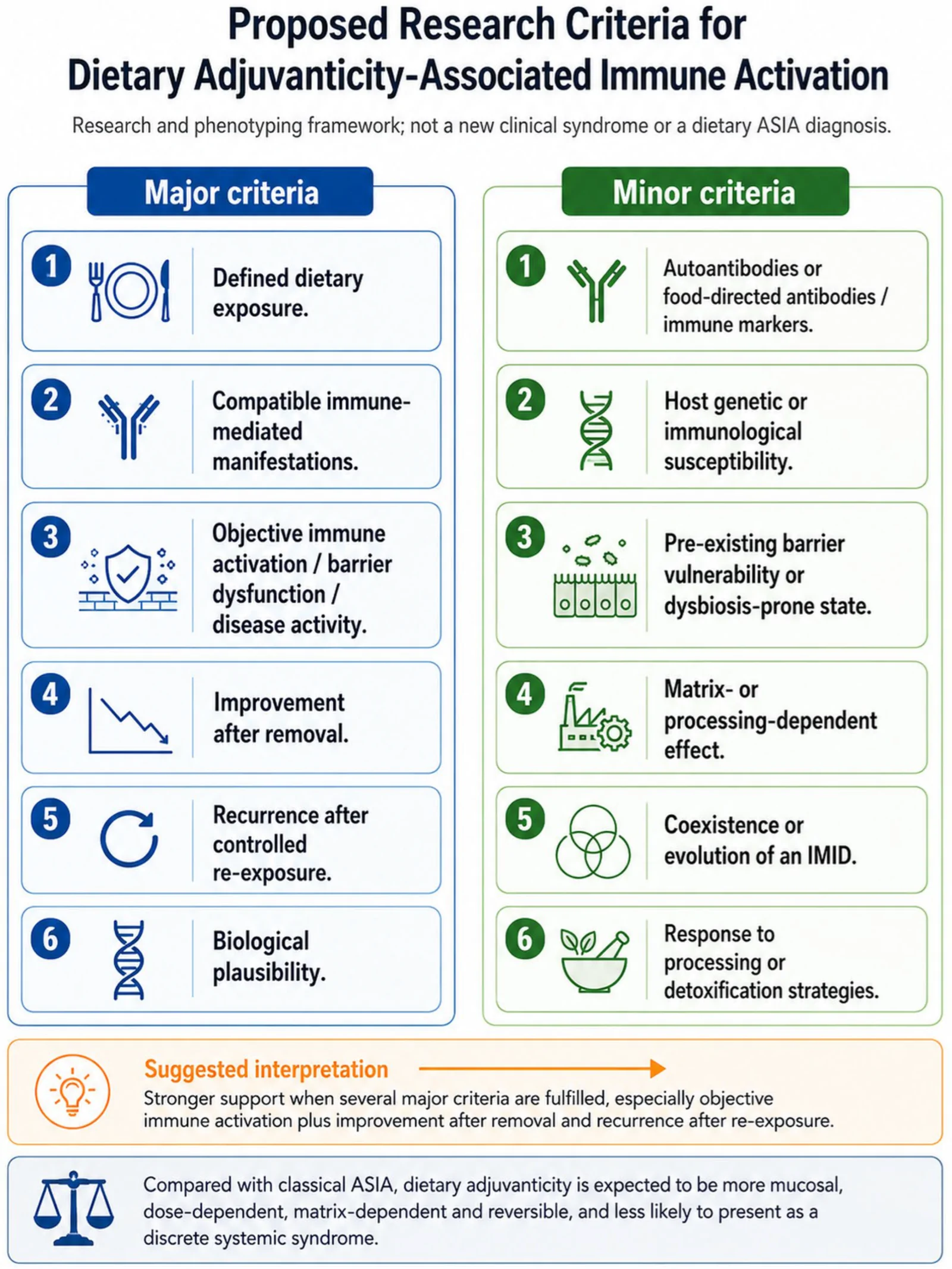
Proposed research criteria for plant/food-derived dietary adjuvanticity-associated immune activation. This framework is intended for research and patient stratification, not as a new clinical syndrome or a dietary ASIA diagnosis.

This distinction helps separate antigenicity from adjuvanticity. In coeliac disease, gluten peptides are disease-defining antigens presented by HLA-DQ2/DQ8 after tissue transglutaminase-mediated deamidation [4,5,6]. Wheat ATIs, by contrast, are not classical coeliac autoantigens, but may amplify innate immune activation and inflammation [28,29,30]. Similarly, potato glycoalkaloids are not autoantigens, yet may affect barrier function and aggravate experimental intestinal inflammation [31,32]. Figure 2 provides research criteria for testing this modifier biology, not a new causal doctrine.

**Table 1:** Candidate plant-derived dietary exposures, structural/biochemical features, and evidence level at the gut barrier-microbiota-host immunity interface.

Exposure/Molecule	Structural/Biochemical Features Relevant to Immune Activity	Main Sources/Matrix	Relevant Mechanism	Disease Context	Evidence Level/Key Refs
Gluten/gliadin epitopes	Proline- and glutamine-rich storage proteins; protease-resistant peptides; tTG-mediated deamidation increases HLA-DQ2/DQ8 binding.	Wheat, rye, barley; wheat-based foods	Antigen-specific adaptive T-cell response and tTG autoimmunity.	Coeliac disease	Established clinical [4,5,6,33]
Wheat ATIs	Small, cysteine-rich pest-resistance proteins; resistant to proteolysis and heat; multiple ATI family members may contribute to activity.	Wheat, barley, rye; processed wheat foods	TLR4-MD2-CD14-related activation of myeloid cells; amplification of intestinal inflammation.	NCWS, IBD models, EAE/MS hypothesis	Strong mechanistic/preclinical; early human translational [28,29,30,34,35,36,37,38]
Wheat germ agglutinin and other lectins	Carbohydrate-binding proteins recognizing glycan motifs; bioactivity depends strongly on structure, dose and processing.	Wheat germ, legumes, cereals	Epithelial and immune-cell interaction through glycan binding; possible barrier/signalling effects.	Barrier and inflammation hypothesis	In vitro/preclinical [39,40,41,42]
Potato glycoalkaloids	Steroidal glycoalkaloids, mainly alpha-solanine and alpha-chaconine; membrane-active amphipathic molecules; increased in greened, sprouted or stressed potatoes.	Potato skins, sprouts, high-glycoalkaloid varieties	Barrier and membrane effects; permeability changes; aggravation of experimental intestinal inflammation.	IBD models	Preclinical [31,32,43]
Quinoa prolamins and saponins	Cultivar-dependent storage proteins; saponins are amphipathic glycosides that can interact with membranes and are affected by washing/processing.	Quinoa grains/flour and gluten-free products	Selected cultivars can activate coeliac-related immune responses ex vivo; matrix and cultivar matter.	Coeliac disease; gluten-free alternatives	Ex vivo/limited [12,13,14,15,44]
Legume proteins and allergens	Seed storage proteins and allergen families; concentration may increase in isolates; cross-reactivity may occur among legumes.	Lupin, pea, lentil, chickpea; plant-protein isolates	IgE-mediated allergy, cross-reactivity, failed oral tolerance; relevance to processed plant-based foods.	Food allergy, EoE, tolerance-failure model	Clinical allergy evidence; indirect for IMIDs [45,46,47,48,49,50]
L-canavanine	Non-protein amino acid structurally related to arginine; may be incorporated or interfere with immune-cell function in susceptible contexts.	Alfalfa sprouts, seeds and supplements	Rare model of plant-derived exposure linked to lupus-like autoimmunity in observations and models.	SLE-like autoimmunity; ASIA-adjacent analogy	Historical clinical/preclinical [51,52,53]
Tolerance-promoting plant substrates	Fermentable fibres, resistant starch, polyphenol-rich matrices, glucosinolates/isothiocyanates; often metabolized by microbiota.	Crucifers, fibre-rich minimally processed foods, fermented/sourdough products	SCFA/Treg pathways, barrier support, antioxidant and anti-inflammatory signalling.	IBD models, uveitis model, digestive prevention	Mostly protective/preclinical and public-health supportive [7,9,11,54,55]

## 5. Crop Breeding, Yield-Oriented Agriculture, and the Changing Plant Exposome

Modern crop breeding has delivered major benefits, including higher yield, food security, pest and disease resistance, improved technological quality, storage stability, and adaptation to intensive agriculture. A balanced discussion must therefore avoid an anti-breeding narrative. In many crops, breeding has intentionally reduced antinutritional or toxic compounds, including low-erucic-acid rapeseed/canola and efforts to reduce phytate, vicine/convicine, ODAP, and other crop-specific compounds [12].

At the same time, conventional breeding and agronomic intensification can have unintended compositional effects. The Lenape potato remains a classic cautionary example: a cultivar selected for chipping quality and resistance was withdrawn after dangerously high glycoalkaloid levels were detected [43]. Disease-resistant celery with high furanocoumarin concentrations was associated with phytophotodermatitis among grocery workers, showing that selection for defence traits may increase human exposure to endogenous plant toxicants [56]. These examples do not show that modern crops are unsafe as a class; they show that plant metabolite profiles can be clinically relevant and should remain part of food-safety assessment.

For IMIDs, the key issue may be less the concentration of one molecule in isolation than the pattern of chronic exposure in a susceptible host. Yield selection, global trade, processing, and plant-based product innovation can change frequency, dose, concentration, and matrix. Gluten-free alternatives, pseudocereals, legume protein isolates, and plant-based meat substitutes have expanded the range of plant antigens and bioactive molecules encountered in Western diets. These exposure shifts are most relevant in diseases where epithelial barrier dysfunction, dysbiosis, and mucosal immune activation are already central [7,8,9].

## 6. Wheat as a Prototype: Gluten, ATIs, and Mucosal Immune Activation

Wheat is the most informative prototype because it contains both disease-defining adaptive immune triggers and non-gluten innate immune activators. In coeliac disease, gluten peptides from wheat, rye, and barley drive HLA-DQ2/DQ8-restricted T-cell responses, with tissue transglutaminase as the major autoantigen and strict gluten avoidance as the core therapy [4,5,6]. This is established clinical evidence for antigenicity, not a speculative adjuvanticity model.

Beyond gluten, wheat contains ATIs, pest-resistance proteins that resist intestinal proteolysis and heat. Junker et al. identified ATIs as potent activators of innate immunity through the TLR4-MD2-CD14 complex, with inflammatory responses in monocytes, macrophages, and dendritic cells [28]. Zevallos et al. subsequently showed that orally ingested ATIs can promote intestinal inflammation through activation of gut and mesenteric lymph node myeloid cells [29]. In mouse models, wheat or wheat ATI consumption aggravated colitis through TLR4 and changed the faecal microbiota [30]. These findings make ATIs one of the strongest examples of a plant-derived dietary adjuvant-like molecule, while still leaving open how broadly this translates to human IMIDs.

**Figure 3:**
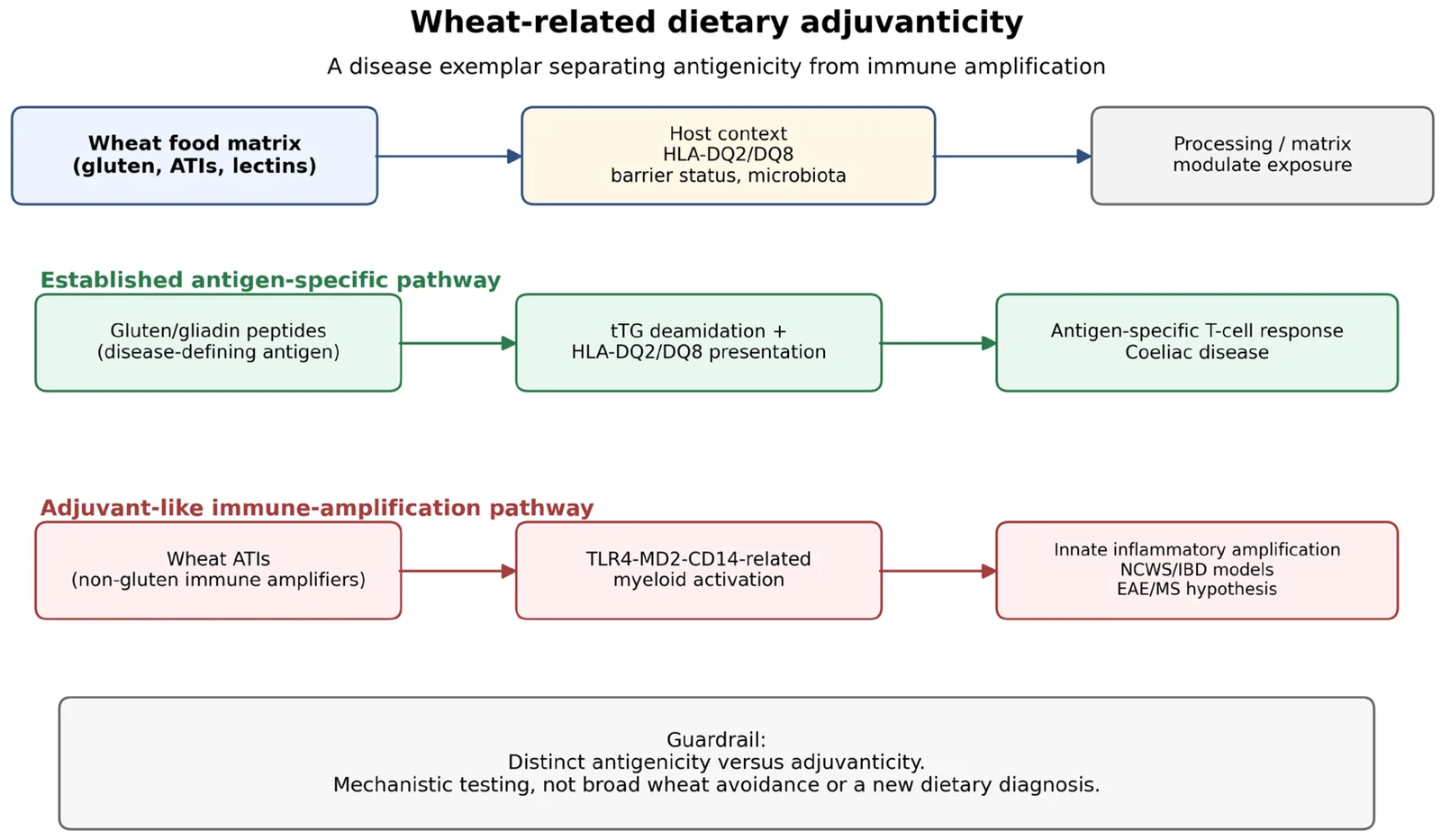
Wheat-related disease exemplar illustrating the distinction between dietary antigenicity and dietary adjuvanticity. Gluten/gliadin peptides represent an established antigenic pathway in coeliac disease, whereas wheat ATIs represent a stronger mechanistic model of adjuvant-like innate immune amplification. The model is intended for mechanistic interpretation and trial design, not for general wheat avoidance recommendations.

The relationship between wheat breeding and immunogenicity is nuanced rather than contradictory. van den Broeck et al. reported differences in coeliac epitopes between old and modern hexaploid wheat varieties and suggested that breeding may have increased epitope exposure [57]. Ribeiro et al., however, did not support a general increase in potential coeliac immunostimulatory epitopes in modern wheat, highlighting substantial inter-variety variation [58]. Geisslitz et al. also found no general increase in ATI content in German wheat bred between 1891 and 2010 [34]. The most defensible conclusion is that modern breeding cannot be described as uniformly increasing wheat immunogenicity. Instead, genotype, breeding history, cultivar, processing, matrix, and host susceptibility shape exposure together (Figure 3).

## 7. Coeliac Disease, Ncws, and Gluten-Free Alternatives

Coeliac disease remains the clearest example of a plant-derived dietary antigen causing a defined immune-mediated disorder in genetically susceptible individuals. The Oslo definitions and recent guidance emphasize gluten ingestion, HLA-DQ2/DQ8 permissiveness, small-intestinal mucosal injury, and coeliac-specific autoantibodies [4,5,6]. It therefore provides a benchmark against which less certain diet-immunity hypotheses should be judged.

NCWS is less clearly demarcated. Symptoms may reflect gluten, ATIs, FODMAPs, or other wheat components, and no single biomarker is accepted for routine diagnosis. Within the dietary adjuvanticity framework, NCWS is useful because it illustrates how plant-derived molecules might provoke symptoms or immune activation outside classical coeliac disease and IgE-mediated allergy. ATIs remain plausible contributors because of their innate immune activity, although clinical translation is still incomplete [28,29,30].

Gluten-free alternatives add a further layer. Quinoa is generally safe and nutritionally useful for patients with coeliac disease, but Zevallos et al. reported cultivar-dependent immune activation: most quinoa cultivars lacked quantifiable coeliac-toxic epitopes, whereas two cultivars activated adaptive and innate immune responses in samples from some patients with coeliac disease [44]. This observation does not undermine quinoa as a gluten-free food. It illustrates that gluten-free does not automatically mean immunologically inert. Cultivar, contamination, processing, matrix, and individual susceptibility may all matter.

## 8. Inflammatory Bowel Disease: Barrier Dysfunction and Dietary Adjuvant-like Molecules

IBD is not driven by a single dietary antigen. It emerges from interactions among genetic susceptibility, the intestinal microbiota, epithelial barrier function, innate and adaptive immunity, and environmental exposures. Diet can influence each component of this network. Western dietary patterns can worsen experimental gut inflammation and are associated with dysbiosis, barrier stress, and mucosal immune activation [7,9].

Against this background, plant-derived adjuvant-like molecules are best considered disease modifiers. Wheat ATIs can aggravate experimental colitis through TLR4-mediated pathways and dysbiosis [30]. Potato glycoalkaloids provide another barrier-relevant example. Patel et al. reported that concentrations encountered during potato consumption can adversely affect intestinal permeability and aggravate experimental IBD [31], and Iablokov et al. found that potato skins containing naturally occurring glycoalkaloids worsened intestinal inflammation in two mouse models [32]. These findings do not support routine potato avoidance in IBD, but they support the principle that endogenous plant toxicants may influence inflammation under susceptible experimental conditions.

In IBD, dietary adjuvanticity should therefore be framed as modifier biology, not primary causation. A patient with active barrier dysfunction, dysbiosis, strictures, post-surgical anatomy, or innate immune sensitivity may respond differently to plant-derived molecules than a healthy person. This may help explain why some patients report food-triggered symptoms without classical allergy or coeliac disease. Future trials should stratify patients by disease activity, microbial profile, barrier markers, medication exposure, and defined dietary exposure rather than treating plant foods as uniform categories.

## 9. Lectins, Protease Inhibitors, Saponins, and Other Plant Defence Molecules

Lectins are carbohydrate-binding proteins present in many plant foods, especially legumes and cereals. Adequate cooking and processing inactivate many lectins, whereas some may retain bioactivity. Wheat germ agglutinin has been shown in epithelial-immune co-culture models to interact with human gastrointestinal epithelial and immune cells [39]. Reviews describe both potentially adverse and potentially beneficial immunomodulatory effects, depending on lectin structure, dose, processing, and context [40,41].

The lectin literature requires careful wording. Popular accounts often overstate links with autoimmune disease, whereas clinical evidence remains limited. A balanced interpretation is that lectins are candidate immune-interacting molecules, not established causes of autoimmunity. Their relevance to dietary adjuvanticity lies in their capacity to bind epithelial or immune-cell glycans, alter signalling, and potentially influence barrier or immune responses in experimental systems [39,40,41].

Other antinutritional factors, including phytate, tannins, oxalates, protease inhibitors, amylase inhibitors, and saponins, also require nuance. These compounds may reduce nutrient bioavailability or interact with digestive and immune pathways, but many also have beneficial pharmacological or microbiome-mediated effects [12,13,14,15]. Glucosinolates and their metabolites from cruciferous vegetables, for example, have been reviewed as potentially beneficial in IBD models through antioxidant, anti-inflammatory, microbiome-modulating, and barrier-restoring effects [54]. Thus, plant defence molecules should not be placed into simple harmful-versus-safe categories; context, dose, processing, and host status are decisive.

Processing is equally important. Soaking, cooking, germination, fermentation, and sourdough processing can reduce or transform phytate, lectins, protease inhibitors, and wheat ATIs [13,33,35,36]. Clinically, the relevant question is rarely whether a plant food is good or bad. It is which molecule, in which matrix, at which dose, and in which host-microbiota context becomes immunologically relevant. Dietary adjuvanticity is therefore a context-dependent property of selected exposures, not an argument against plant-rich diets.

### Protective and Tolerance-Promoting Plant Exposures

The plant exposome should not be framed as intrinsically harmful. Many plant-derived substrates support epithelial integrity, microbial diversity, and immune tolerance. Fermentable fibres promote short-chain fatty acid production, while polyphenol-rich foods, resistant starches, and diverse minimally processed plant matrices may support anti-inflammatory microbial functions and barrier resilience [7,9,11,55]. Similarly, glucosinolate-derived metabolites from cruciferous vegetables may be protective in IBD models through antioxidant, anti-inflammatory, barrier-restoring, and microbiome-modulating pathways [54]. This protective counterpoint is essential: the proposed framework asks when selected exposures become relevant, not whether plant foods should be avoided.

## 10. Novel Plant Foods and Emerging Plant Protein Exposures

Globalized diets have introduced, or markedly increased, exposure to plant foods that were once uncommon in many Western populations. Examples include quinoa, amaranth, buckwheat, chia, sesame, lupin, and concentrated proteins from pea, lentil, and chickpea. Many are nutritionally valuable, environmentally attractive, and useful in gluten-free or plant-based diets. Immunologically, however, they also represent new or newly concentrated antigenic exposures.

Food allergy provides the clearest clinical evidence that novel plant exposure can be relevant. Non-priority legumes, including pea, chickpea, lentil, and lupin, are increasingly recognized as allergen sources capable of reactions ranging from mild symptoms to anaphylaxis [45,46,47]. Lupin is important because it is used in gluten-free bakery products and may cross-react with peanut allergy; it has been described as both a new and hidden food allergen [48]. Buckwheat can cause IgE-mediated allergy and anaphylaxis in settings where intake is increasing [59]. Sesame is now recognized globally and has been added as a major food allergen for labelling in the United States [60].

The connection between these allergens and autoimmune IMIDs is indirect. IgE-mediated food allergy is not IBD, coeliac disease, or systemic autoimmunity. Even so, allergy demonstrates a broader principle: plant proteins introduced into a population, or presented in concentrated processed forms, can become clinically meaningful immune exposures. Plant-based meat analogues and protein isolates therefore warrant allergenomic and immunological evaluation beyond macronutrient profiling [49,61].

### Food-Level Examples: From Molecules to Modern Food Matrices

At the food level, dietary adjuvanticity is most relevant to specific molecules, cultivars, matrices, and processing contexts rather than to broad food categories (Table 2). Wheat-based foods provide exposure to gluten epitopes, ATIs, and wheat lectins. Potato skins and greened or sprouted potatoes may carry higher glycoalkaloid loads. Novel gluten-free or plant-based products may introduce quinoa cultivars, lupin flour, pea protein isolates, and other concentrated legume proteins into groups that previously consumed them less often. Alfalfa sprouts or supplements are a striking example: L-canavanine has been linked to lupus-like autoimmunity in human observations, primate models, and immunological studies [51,52,53]. These examples do not make the foods intrinsically harmful; they show why molecule, dose, processing, microbiome context, and host susceptibility should be evaluated together.

## 11. Systemic and Extraintestinal IMIDs: Plausible Extensions of Dietary Adjuvanticity

Dietary adjuvanticity is not limited to disorders in which the gut is the clinically dominant organ. The intestine is a large immune interface where plant molecules, microbes, metabolites, and host immune cells meet. Mucosal immune activation can influence systemic inflammation through microbial products, metabolites, cytokines, trafficking immune cells, and shared barrier pathways. This does not mean that plant-derived molecules cause systemic autoimmunity; it means that selected exposures may modify diseases in which gut-immune connections are already biologically plausible.

### 11.1. Multiple Sclerosis and Neuroinflammation

Multiple sclerosis (MS) is currently the strongest extraintestinal example because it links a defined plant-derived molecule with a model of organ-specific autoimmunity. Dietary wheat ATIs exacerbated central nervous system inflammation in experimental autoimmune encephalomyelitis, activated intestinal myeloid cells, and promoted inflammatory mediators in monocytes from patients with MS and controls [37]. In a small open-label crossover pilot trial in relapsing-remitting MS, a wheat- and ATI-reduced diet did not meet the primary T-cell endpoint but was associated with changes in monocyte subsets and improved pain-related quality of life [38]. These data do not justify general wheat restriction in MS. They provide a coherent example of a gut-derived dietary immune amplifier that may influence a distant IMID and deserves controlled clinical testing.

### 11.2. Rheumatoid Arthritis and Spondyloarthritis: The Gut-Joint Axis

RA and SpA are useful because both can be viewed through a gut-joint lens. In RA, dysbiosis, altered intestinal barrier function, and diet-sensitive microbial metabolites have been linked to immune regulation, Th17/Treg balance, and disease activity, although causality is difficult to prove [62]. In SpA, the overlap with IBD, subclinical gut inflammation, dysbiosis, and IL-23/IL-17 biology provides a stronger mucosal rationale [63]. Here, dietary adjuvanticity should be treated as a testable modifier hypothesis. ATIs, lectins, saponins, or glycoalkaloids are not plausible disease-specific autoantigens for RA or SpA, but they may influence the mucosal inflammatory set point in patients whose disease already involves gut-immune crosstalk.

### 11.3. Psoriasis and Psoriatic Arthritis: The Gut-Skin-Joint Axis

Psoriasis and PsA offer another extension of the model. Reviews increasingly emphasize the gut-skin axis, in which dysbiosis, microbial metabolites, inflammatory mediators, and barrier integrity may influence cutaneous immune responses [64]. In PsA, a gut-skin-joint axis has been proposed to connect microbiome alterations with systemic inflammation and joint disease; dietary or microbiome-directed approaches are discussed as adjunctive rather than primary disease treatments [65]. In this setting, dietary adjuvanticity is best framed as gut-derived inflammatory amplification that could modulate IL-17/TNF-driven pathways.

### 11.4. Autoimmune Thyroiditis and Type 1 Diabetes

Autoimmune thyroiditis and type 1 diabetes (T1D) are relevant for different reasons. Thyroid autoimmunity appears in the ASIA literature as part of the endocrine autoimmune spectrum [66,67]. By contrast, evidence for gluten-free diets in non-coeliac Hashimoto’s thyroiditis remains inconsistent: a recent systematic review and meta-analysis reported very low-certainty evidence, no significant effect on TSH or thyroid hormones, and divergent effects on thyroid antibodies [68]. Thus, wheat or gluten-related exposures may matter in selected patients, especially with coeliac disease, but generalized gluten avoidance is not justified. In T1D, preclinical and microbiome data are more suggestive. NOD mice raised on gluten-free diets had lower spontaneous diabetes incidence with altered intestinal microbiota, and adding gluten reversed the protective effect [69]. A 2026 review also frames diet as a driver of microbiome development and a potential modulator of T1D risk and progression [70]. These observations support a gluten-microbiome-immune axis, but not yet a clinical dietary adjuvanticity intervention in T1D.

### 11.5. Autoimmune Uveitis and Ocular Inflammation

Autoimmune uveitis provides a useful proof of principle that gut-directed dietary interventions can affect a distant immune-mediated organ. In an experimental autoimmune uveitis model, a fermentable-fibre-rich diet altered the microbiota, improved intestinal homeostasis and permeability, increased regulatory T-cell responses, and reduced ocular inflammation [55]. This example also keeps the framework balanced: not all plant-derived exposures are pro-inflammatory. Fermentable fibres and polyphenol-rich foods may strengthen tolerance pathways. Dietary adjuvanticity should therefore be understood as a property of selected molecules in specific contexts, not as a feature of plant foods as a whole.

### 11.6. Food Allergy, Eosinophilic Oesophagitis, and Concentrated Plant Protein Exposures

Food allergy and EoE are not autoimmune diseases, but they are clinically important models of failed immune tolerance to dietary antigens. They are also directly relevant to modern plant-protein exposure. Lupin, pea, lentil, and chickpea proteins are increasingly used in gluten-free and plant-based foods, and may appear as hidden allergens or concentrated protein isolates [45,46,47,48]. The updated ACG guideline for EoE highlights food-antigen-driven disease and diet as a core management domain [50]. This supports a cautious extension of the framework: concentrated plant proteins can be clinically meaningful immune exposures, even when the mechanism is allergic or eosinophilic rather than autoimmune.

### 11.7. Interpretation of the Expanded Disease Spectrum

Across these extraintestinal examples, the evidence is uneven. The ATI-EAE/MS literature provides the most direct mechanistic support. RA, SpA, psoriasis/PsA, T1D, and uveitis provide biologically plausible gut-immune models, but not disease-specific proof for plant molecules. Food allergy and EoE provide strong clinical evidence for plant-protein immune reactions, although they remain mechanistically distinct from autoimmunity. These disease-level extensions are summarized in Table 3 and should be read as hypothesis-generating rather than practice-changing.

## 12. Clinical and Public-Health Implications

Clinical implications must remain conservative. Current evidence does not support generalized elimination of wheat, legumes, nightshades, cruciferous vegetables, or novel plant foods for all patients with IMIDs. Such advice could reduce nutritional adequacy, narrow dietary diversity, and increase food-related anxiety. Clinicians should distinguish established diagnoses, such as coeliac disease or IgE-mediated food allergy, from broad avoidance strategies that remain unproven.

At the population level, this disease-modifier framework should align with established digestive-health prevention policies rather than narrow food avoidance. UEG recommendations emphasize structural measures such as harmonized front-of-pack labelling, fiscal policies for sugar-sweetened beverages and high-sugar or high-salt ultra-processed foods, reformulation targets, marketing restrictions to protect children, and better access to affordable minimally processed foods [11].

For coeliac disease, strict lifelong gluten avoidance remains evidence-based; the immunological safety of gluten-free alternatives should be considered in relation to contamination, cultivar variability, and symptoms [4,5,6,44]. In IBD, diet should be individualized according to disease activity, nutritional status, strictures, resections, intolerance patterns, and objective inflammation. In food allergy, hidden plant allergens such as lupin, sesame, pea, and buckwheat should be actively considered, particularly in gluten-free and plant-based processed foods [45,48,59,60].

For extraintestinal IMIDs, the message should be even more restrained. Current evidence does not support broad avoidance of wheat, legumes, or plant-based foods in MS, RA, SpA, psoriasis, autoimmune thyroiditis, T1D, or uveitis. Conventional exceptions remain clear: gluten avoidance in coeliac disease, avoidance of proven allergens, individualized EoE management, and carefully supervised dietary trials when clinically justified. At present, dietary adjuvanticity is mainly a conceptual and investigational tool for generating mechanistic hypotheses and defining trial subgroups.

## 13. Integrative Synthesis and Evidence Balance

The evidence reviewed here supports a graded interpretation. At the strongest end, gluten in coeliac disease and IgE-mediated allergy to selected plant proteins represent established clinical models of food-driven immune disease. At the mechanistic level, wheat ATIs provide the most convincing prototype of plant-derived adjuvant-like activity because receptor-related myeloid activation, intestinal inflammation, and experimental disease amplification have been demonstrated [28,29,30,37]. At a lower evidence level, lectins, glycoalkaloids, quinoa prolamins, saponins, and L-canavanine illustrate how plant-derived molecules may interact with epithelial barriers, immune cells, or disease-prone contexts, but their relevance to common human IMIDs remains incompletely established [31,32,39,40,41,42,44,51,52,53,54].

The main scientific contribution of this review is therefore conceptual rather than prescriptive. Dietary adjuvanticity integrates antigenicity, barrier dysfunction, innate immune activation, microbiota-dependent transformation, processing, and host susceptibility into a testable framework. It also creates an operational distinction between disease-defining dietary antigens and disease-modifying dietary immune amplifiers. This distinction is important because it prevents overextension: a molecule may amplify mucosal inflammation without being an autoantigen or a sufficient cause of disease.

The framework also has negative boundaries. It does not imply that plant foods are generally harmful, that modern crops are broadly dangerous, that ASIA can be diagnosed from dietary symptoms, or that patients with IMIDs should adopt unsupervised exclusion diets. Many plant-derived substrates are protective or tolerance-promoting. The practical task is to identify defined exposures, susceptible subgroups, processing conditions, and biomarkers that convert a broad hypothesis into testable human studies.

## 14. Future Directions

Future work should prioritize methodological validation. Candidate exposures should be chemically and structurally defined, including protein family, peptide motif, glycosylation or glycan-binding properties, alkaloid or saponin content, cultivar, processing history, dose, matrix, and microbial transformability. Functional testing should combine epithelial barrier models, organoids, dendritic-cell and macrophage assays, PRR signalling assays, cytokine profiling, and microbiome/metabolome readouts.

Human studies should move beyond broad food categories. Intervention trials should stratify participants by genotype, disease activity, barrier markers, medication exposure, previous diet, microbial profile, and symptom/inflammation phenotype. Outcomes should include objective markers of mucosal inflammation, permeability, microbial metabolites, immune activation, and validated clinical endpoints. Trials should distinguish whole foods from purified components, raw from processed matrices, and absolute from relative exposure.

Extraintestinal IMIDs offer a promising but demanding testing ground. ATI-reduced dietary interventions in MS, gut-joint studies in RA and SpA, gut-skin-joint studies in psoriasis/PsA, and barrier-focused studies in autoimmune uveitis or T1D should include immune phenotyping, intestinal permeability markers, targeted microbiome/metabolome profiling, and pre-specified clinical endpoints. Such studies should avoid broad dietary ideology and test defined molecules, defined patient subgroups, and defined mechanisms.

Food technology should become an active part of this research agenda. Future studies should not only identify plant-derived immune-active molecules, but also define how processing conditions modify their biological activity. Thermal treatment, fermentation, enzymatic hydrolysis, extrusion, high-pressure processing, irradiation, milling intensity, particle size, and matrix formulation may influence protein conformation, glycan accessibility, protease resistance, epithelial bioaccessibility, microbial transformation, and ultimately mucosal immune recognition. For candidate molecules such as wheat ATIs, lectins, glycoalkaloids, saponins, prolamins, and concentrated legume proteins, systematic processing studies could help determine which technological parameters reduce adjuvant-like activity or intestinal exposure without compromising nutritional value. Such interventions should be evaluated carefully, because processing may occasionally generate new epitopes, increase bioavailability, or shift microbial metabolism in unexpected ways.

At the food-system level, dietary adjuvanticity should be studied alongside exposure distribution. Product formulation, affordability, marketing, labelling, and access to minimally processed foods may determine which populations encounter concentrated plant proteins, novel allergens, or altered matrices [10,11]. Thus, validation requires collaboration between food technologists, immunologists, gastroenterologists, allergologists, microbiome scientists, public-health experts, and crop scientists.

## 15. Limitations

This review is hypothesis-generating and integrates heterogeneous evidence. ASIA remains debated, and its use here is explicitly analogical. The plant-derived exposures discussed differ in structure, dose, processing sensitivity, microbial transformability, and clinical relevance. Evidence is strong for gluten in coeliac disease and for IgE-mediated allergy to several plant proteins. Mechanistic evidence is substantial for wheat ATIs, but clinical translation remains incomplete. For lectins, glycoalkaloids, quinoa prolamins, L-canavanine, and many novel foods, human IMID data are limited. The framework should therefore stimulate research rather than drive broad dietary restriction.

A further limitation is that the beneficial effects of plant foods must be preserved in clinical interpretation. Fibre, polyphenols, glucosinolates, resistant starch, and diverse plant-based diets may support microbiome diversity and anti-inflammatory metabolite production. The aim is not to discourage plant consumption, but to identify specific molecules that may become relevant in specific susceptible hosts or disease states.

The extraintestinal sections add another layer of uncertainty. In most systemic IMIDs, current evidence supports gut-immune plausibility rather than plant-molecule causality. The strongest exception is the ATI-EAE/MS literature, which still requires larger controlled clinical trials. These sections should therefore be read as hypothesis-generating extensions of the dietary adjuvanticity framework.

## 16. Conclusions

Dietary adjuvanticity is proposed here as a specific, testable framework for studying how selected food-derived molecules may amplify mucosal immune responses at the gut barrier-microbiota-host immunity interface. The novelty of the framework lies in separating dietary antigenicity from adjuvant-like immune amplification, while integrating food matrix, processing, microbiota, barrier function, and host susceptibility into one disease-modifier model.

The strongest established clinical model remains gluten-driven coeliac disease, whereas wheat ATIs provide the clearest mechanistic example of plant-derived innate immune amplification. Other candidate exposures, including lectins, glycoalkaloids, quinoa prolamins, saponins, L-canavanine, and concentrated legume proteins, require more cautious interpretation because much of the evidence remains preclinical, ex vivo, allergological, or indirect. The ASIA concept provides a useful analogy for exogenous immune amplification, but it should not be used to diagnose dietary reactions or to claim that plant foods cause ASIA syndrome.

The translational message is therefore conservative but important. Plant-derived exposures may be protective, neutral, or pro-inflammatory depending on molecule, matrix, processing, microbiota, disease activity, and host genetics. The framework does not support indiscriminate plant-food avoidance. It supports more precise research into defined exposures, susceptible patient subgroups, immune and barrier biomarkers, and food-technological strategies that may reduce undesirable immune activity while preserving nutritional value.

## Figures and Tables

**Table 2:** Food-level examples of dietary adjuvanticity-relevant exposures and processing contexts.

Food or Product Example	Relevant Exposure	Why It Fits the Framework	Immune/Disease Context	Key Refs
Wheat bread, durum pasta, pizza, bulgur, couscous, seitan; wheat germ/whole-grain products	Gluten epitopes; wheat ATIs; wheat lectins	Food matrix combining established antigenicity, non-gluten innate immune activation and epithelial/lectin interactions.	Coeliac disease, NCWS, IBD models, MS/EAE; barrier hypothesis	[4,5,6,28,29,30,34,35,36,37,38,39,40,41,42]
Potato skins, greened/sprouted potatoes, high-skin potato products	Alpha-solanine, alpha-chaconine and related glycoalkaloids	Storage- and matrix-dependent exposure to barrier-relevant plant toxicants.	IBD models; intestinal permeability	[31,32,43]
Alfalfa sprouts, seeds and supplements	L-canavanine	Rare but striking plant-derived exposure linked to lupus-like autoimmunity in observations and models.	SLE-like autoimmunity; ASIA-adjacent analogy	[51,52,53]
Quinoa grains/flour and quinoa-containing gluten-free products	Quinoa prolamins; saponins	Cultivar-dependent immune responses among gluten-free alternatives.	Coeliac disease; gluten-free food safety	[12,13,14,15,44]
Lupin flour in gluten-free bakery products and processed foods	Lupin proteins	Hidden allergen; cross-reactivity with peanut allergy; increasingly used in high-protein/gluten-free foods.	Food allergy; failed dietary tolerance model	[45,46,47,48,49]
Pea protein isolates; chickpea/lentil concentrates; legume pasta; vegan burgers/bars	Concentrated legume proteins; residual lectins/protease inhibitors	Modern processing may intensify plant-protein exposure beyond traditional patterns.	Food allergy, EoE, novel plant antigen exposure	[45,46,47,49,50,61]
Buckwheat noodles/flour; sesame-rich foods such as tahini and hummus	Buckwheat and sesame allergens	Globalized plant foods with recognized allergic potential in Western diets.	Food allergy; tolerance-failure model	[49,59,60]
Ultra-processed plant-based foods and high-protein snacks	Concentrated proteins, additives, altered matrix	Food-system example linking processing, UPF exposure, additives, dysbiosis and barrier stress.	Digestive health, IBD/IBS/CRC prevention context	[10,11,49,61]
Cruciferous vegetables; fermented/sourdough plant foods	Glucosinolates/isothiocyanates; reduced antinutrients; microbial metabolites	Counter-example: plant exposures may promote tolerance and barrier resilience.	Potentially protective microbiota-barrier-host context	[13,14,15,35,36,54]

**Table 3:** Possible extension of dietary adjuvanticity to extraintestinal immune-mediated diseases.

Disease Context	Rationale for Inclusion	Candidate Exposure/Mechanism	Evidence Strength/Key Refs	Recommended Framing
MS/EAE	Defined gut-brain immune model with ATI data	Wheat ATIs; TLR4-related myeloid activation; intestinal-to-CNS inflammatory amplification	Mechanistic/preclinical plus small pilot clinical trial [37,38]	Most direct extraintestinal extension; no general dietary recommendation yet
RA	Gut-joint axis; dysbiosis, barrier and diet-sensitive microbial metabolites	Mucosal inflammatory set point; Th17/Treg balance; microbiome-mediated immune modulation	Indirect/moderate [62]	Potential disease modifier; specific plant molecules remain untested
SpA	Overlap with IBD, subclinical gut inflammation and IL-23/IL-17 biology	Barrier dysfunction, dysbiosis, mucosal immune activation	Indirect/moderate [63]	Gut-joint modifier hypothesis
Psoriasis/PsA	Gut-skin-joint axis and systemic inflammatory pathways	Microbial metabolites, barrier integrity, IL-17/TNF inflammatory amplification	Indirect/moderate [64,65]	Gut-derived inflammatory amplifier, not disease-specific trigger
Autoimmune thyroiditis	ASIA endocrine autoimmunity literature; debated gluten/GFD data	Possible gluten/wheat relevance in selected patients; coeliac overlap	Controversial/low certainty [66,67,68]	No generalized gluten-free recommendation without indication
T1D	Gluten/NOD mouse data and diet-microbiome-immune axis	Gluten-related microbiome changes; barrier and immune modulation	Preclinical plus observational context [69,70]	Plausible but not clinical guidance
Autoimmune uveitis	Experimental evidence that dietary fibre can affect gut permeability and ocular inflammation	Fermentable fibre, SCFA/Treg pathways; barrier restoration	Preclinical proof-of-principle [55]	Shows bidirectionality: plant exposures can also be tolerance-promoting
Food allergy/EoE	Clinical immune reactions to dietary antigens; emerging plant protein exposures	Lupin, pea, lentil, chickpea and other concentrated plant proteins; IgE and non-IgE mechanisms	Strong for allergy; indirect for autoimmunity [45,46,47,49,50]	Useful tolerance model, mechanistically distinct from IMIDs

## Data Availability

No new data were created or analyzed in this study. Data sharing is not applicable to this article.

## Abbreviations

ACG, American College of Gastroenterology; ASIA, autoimmune/inflammatory syndrome induced by adjuvants; ATI, amylase-trypsin inhibitor; CNS, central nervous system; CRC, colorectal cancer; EAE, experimental autoimmune encephalomyelitis; EoE, eosinophilic oesophagitis; HLA, human leukocyte antigen; IBD, inflammatory bowel disease; IBS, irritable bowel syndrome; IFN, interferon; IL, interleukin; IMID, immune-mediated inflammatory disease; MS, multiple sclerosis; NCWS, non-coeliac wheat sensitivity; NLR, NOD-like receptor; NOD, non-obese diabetic; ODAP, beta-N-oxalyl-L-alpha,beta-diaminopropionic acid; PRR, pattern-recognition receptor; PsA, psoriatic arthritis; RA, rheumatoid arthritis; SCFA, short-chain fatty acid; SLE, systemic lupus erythematosus; SpA, spondyloarthritis; T1D, type 1 diabetes; TLR, Toll-like receptor; TNF, tumour necrosis factor; Treg, regulatory T cell; tTG, tissue transglutaminase; UEG, United European Gastroenterology; UPF, ultra-processed food.
